# Utilizing Impedance for Quality Assessment of European Squid (*Loligo Vulgaris*) during Chilled Storage

**DOI:** 10.3390/foods8120624

**Published:** 2019-11-29

**Authors:** Sandra Zavadlav, Igor Lacković, Danijela Bursać Kovačević, Ralf Greiner, Predrag Putnik, Sanja Vidaček Filipec

**Affiliations:** 1 Karlovac University of Applied Sciences, Trg J. J. Strossmayera 9, 47 000 Karlovac, Croatia; 2 University of Zagreb, Faculty of Electrical Engineering and Computing, Unska 3, 10 000 Zagreb, Croatia; igor.lackovic@fer.hr; 3 University of Zagreb, Faculty of Food Technology and Biotechnology, Pierottijeva 6, 10 000 Zagreb, Croatia, svidacek@pbf.hr (S.V.F.); 4 Department of Food Technology and Bioprocess Engineering, Max Rubner-Institut, Federal Research Institute of Nutrition and Food, Haid-und-Neu-Straße 9, 76131 Karlsruhe, Germany; ralf.greiner@mri.bund.de

**Keywords:** squid, *Loligo vulgaris*, bioelectrical impedance, chilled storage, storage days, quality

## Abstract

This study evaluates the quality of chilled squid *Loligo vulgaris* by non-destructive measurements of bioelectrical impedance from the first post-mortem day under controlled conditions. Squid samples were stored at 4.5 °C and 55% of relative humidity for 11 days. Impedance magnitude (|Z|) and phase (φ) at 200 frequencies (100Hz to 100MHz) were measured using an Agilent 4294A Precision Impedance Analyzer with needle-type multi-electrode array on days 1, 2, 3, 4, 5, 8, 9, 10, and 11 of storage. The changes in color, sensory properties, total volatile nitrogen, pH, and water holding capacity were also determined. The obtained results indicated that the samples could be classified into five to six distinctive groups by measuring the electrical parameters at frequencies close to 5MHz. In general, φ is less dependent on temperature and measurement setup than |Z|, while records at 5MHz correlate well with the days of storage (*R*^2^ = 0.968). The data imply that it is only possible to estimate the length of storage for the samples with measurements of phase angle, which can be useful for the development of new analytical instruments. Biosensors have a practical industrial application, as it is demonstrated that bioelectrical impedance data correlates well with the days of chilled storage.

## 1. Introduction

Global seafood consumption has risen continuously over the last fifty years, and seafood is a highly traded food over the entire globe by complex distribution channels. The temperature of fish in a supply-chain often fluctuates, which results in rapid loss of freshness, increased bacterial growth, and shorter shelf life. Rapid, instrumental, and non-destructive measurements of quality, freshness, and storage can improve processing and trade, generate less discards in stores and homes, and hence support sustainable food trades [1]. Therefore, the development and testing of methods for the detection of changes in seafood post-mortem, i.e., from time of catch until the expiry date, has been researched for decades. Loss of freshness (e.g., autolytic and enzymatic reactions) and gradual occurrence of spoilage (e.g., microbial activity) can be determined by sensory, physical, chemical, biochemical, microbiological, and electrical methods. Such methods have been developed by various stakeholders in the food chain [2,3]. However, three key techniques are traditionally used for assessing storage and spoilage of marine organisms, namely, sensory assessment, chemical analysis (including Total Volatile Basic Nitrogen (TVB-N)), and microbial evaluation.

The European Union Freshness Grading (EU or EC Scheme) Freshness Grades for Fishery Products (or the EC scheme) is the only guidance for the assessment of freshness in fishery products. Sometimes also referred to as the “quality index method (QIM)”, based on a structured scaling for quality measurements, it can be used for the prediction of the remaining shelf life, but it is not applicable for European squid. The incompatibility of the system is due to its exclusive dedication to cephalopods, e.g., the species of cuttlefish *Sepia officinalis* and *Rossia macrosoma* and some other fishery products [4,5]. Although the EC scheme determines the freshness, the point-of-storage of fishery products (from catch to the point of analysis) is based on species-specific sensory approaches. A significant parameter for the assessment of seafood quality and one of the most common chemical indicators of spoilage is referred to as total volatile basic amines (TVB-N) [6,7,8]. In particular, TVB-N can be useful for the quality assessment in cephalopods such as squid [9]. According to the EU directive for fish hygiene, food inspectors must use TVB-N for chemical checks if the organoleptic assessment is doubtful regarding the freshness of the fish [10]. However, TVB-N detects (at least in fish) later stages of quality deterioration during storage [11] whereas cephalopods muscles have the tendency to remain microbiologically less spoiled [4]. Other parameters, as color changes [12,13], the content/concentration of free amino acids [14], and protein solubility [15,16,17] are all well correlated with spoilage in squids, hence are potential indicators of spoilage.

One of the methods potentially useful for quality control in seafood is bioelectrical impedance [18]. Biological materials have both resistive and capacitive properties [19] that change with animal death and post-mortem. Therefore, it is possible to develop tools for assessing the storage time after catch. Bioelectrical impedance is useful for the development of on-line detectors that are able to replace labor-intensive traditional methods (demanding of time, costs, skilled personnel, and additional quality grading). Current research about fishery products is focused on utilizing dielectric properties and relating them to the quality of fish and other seafood [20,21,22,23]. It is long-known that those dielectric properties are linked to the structure or chemical composition of tissues [24]. Furthermore, our previous results have revealed a high correlation between electrical parameters and post-mortem changes in the protein structures of cephalopods [15]. Additionally, the electrical parameters align with the spoilage of the second phase, i.e., post rigor mortis in *L. vulgaris* stored at 4 °C. By using phase φ at frequencies close to 1 MHz, the samples used in the study were grouped into three distinctive categories, while electric properties were associated with protein deterioration. These findings are a good basis for employing dielectric properties as quality indicators for monitoring chilled storage of cephalopods.

During the electrical measurements of muscular tissues, several parameters such as electrode polarization, temperature dependence, and anisotropy of the tissues should be considered. For the measurements of |Z| and φ in chilled *L. vulgaris*, the transversal direction of the electrode to the circumferential muscle fibers was selected as the best option. That position of the array improved differentiation of storage time among the samples between day 4 (after rigor mortis) and day 15 [15]. The obtained results could pave the way for the development of a simple, rapid, and non-expensive instrumental assessment to measure the duration of storage (days) of chilled European squids. Therefore, the aim of this research is to evaluate the reliability of electrical impedance at frequencies of up to 100 Hz as indicators for quality changes in European squid (*L. vulgaris*) stored at 4.5 °C and 55% relative humidity for 11 days. During the study, data were collected for changes of color, sensory properties, total volatile nitrogen, pH, and water holding capacity.

## 2. Materials and Methods

### 2.1. Squid Samples

**Figure 1:**
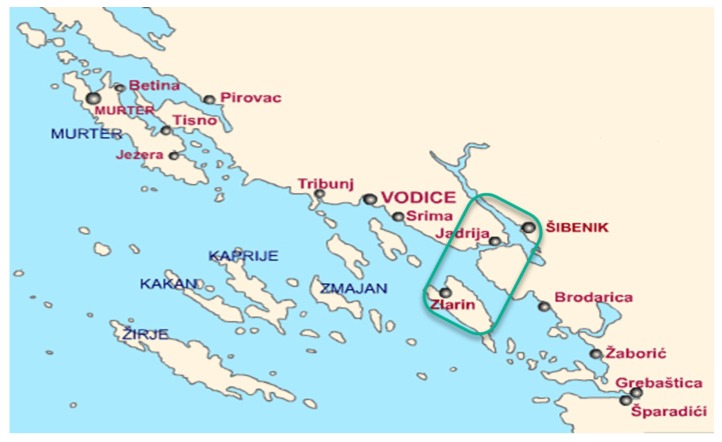
Area of fishing between the town of Šibenik and the island Zlarin (Croatian Adriatic coast).

European squids (*Loligo vulgaris*, Lamarck 1798) were caught by one net during a single night between the town of Šibenik and the island of Zlarin (Figure 1; *n* = 72). The samples were transported to the laboratory in Styrofoam boxes packed with crushed ice. Upon arrival, the entire squids (average *m* = 130 ± 20 g) were randomly divided into 9 groups (*n* = 8 per group) and stored under controlled temperature and humidity (T = 4.5 °C; relative humidity of 55%) until analysis. Each unit of a sample was wrapped in low-density polyethylene (LDPE) films (225 micron thick) to minimize losses of humidity. Temperature and humidity were recorded by thermo-hygro meters (Model 13307, Thermo-Hygrometer, DeltaTRAK, Pleasanton, CA, USA). The mantle length of squids averaged 16.5 ± 0.5 cm, with a thickness of 0.3 ± 0.05 cm, and a width of 4.2 ± 0.3 cm. The samples were analyzed (impedance, skin color, and sensory evaluation) on days 1, 2, 3, 4, 5, 8, 9, 10, and 11 post-mortem. According to the literature, the expected shelf life of the squids is 12–14 days. TVB-N, pH, and water holding capacity (WHC) were observed on days 2, 5, 9 and 11. The measurements of impedance took place on day 1 after fishing when the samples had rigor mortis. Squid samples were whole, meaning they were not skinned, decapitated, and gutted before any analysis.

### 2.2. Electrical Impedance Spectroscopy Analysis

Electrical impedance spectroscopy was used to determine electrode impedance using an Agilent 4294A precision impedance analyzer controlled by TCP/IP protocols (Santa Clara, CA, USA). A custom-designed needle-type multi-electrode array consisting of two rows with six parallel, electrically connected, gold-plated needles was connected to the Agilent 16047E test fixture to provide a bipolar measurement set-up. The rationale behind this electrode setup has been thoroughly elaborated in literature [15,25,26,27,28]. Impedance magnitude |Z| and phase (φ) were measured at 200 various frequencies between 100 Hz and 100 MHz.

Before conducting impedance measurements, parasitic impedances were corrected by “open” and “short” compensations. In order to achieve the electrode polarization (below 5 kHz and reduced at higher frequencies), impedance was taken with the array in 50 and 142 mM NaCl solutions (*m*/*v*). The array was introduced into the muscular fibers of a mantle at a 90° angle with full insertion into the samples. The array was orthogonal to the longitudinal axis, e.g., transversal to the direction of the circumferential bands on the ventral part of the mantle [15]. The temperature of the samples was monitored by two thermometers (Model 26000, Heat⁄Cool Thermometer DeltaTRAK, and Model 13307, Thermo-Hygrometer, DeltaTRAK, Pleasanton, CA, USA). Measurements were performed in triplicates for each squid sample at T = 6 ± 1 °C.

### 2.3. Measurement of Color Parameters

Color parameters were measured with a Minolta CM-700d spectrophotometer (Osaka, Japan) with the target mask CM-A178 (diameter 8 mm). The L* (lightness), a* (redness), and b* (yellowness) were recorded using a D65 as a light source. Prior to analysis, the spectrophotometer was calibrated with white calibration cap CM-A177. For each squid sample, the color was measured at six positions on the mantle dorsal surface at T = 6 ± 1 °C.

### 2.4. Determination of Total Volatile Base Nitrogen, pH and Water Holding Capacity

TVB-N was determined according to [29]. pH was determined with a pH-meter (Sartorius AG, Scientific, Goettingen, Germany) according to [30]. Briefly, five grams of homogenized sample were mixed with five volumes of distilled water (*w*/*v*), and pH was determined. Water holding capacity was determined according to [31].

### 2.5. Sensory Analysis

Sensory evaluation of the raw chilled squids (*Loligo vulgaris*) was done by adequately trained panelists (*n* = 15 experts) based on the Guide to EC freshness grades [32].

### 2.6. Statistical Analysis

Data distribution was evaluated by the Kolmogorov-Smirnov and Shapiro-Wilk tests, while Levene’s test assessed the homogeneity of variances. ANOVA and Tukey’s tests evaluated the variance in the dataset, while correlation among variables was evaluated by Pearson’s correlation. Significance for all tests was *p* ≤ 0.05, and SPSS version 9.0 (IBM, Chicago, IL, USA) was used for data analysis.

## 3. Results and Discussion

### 3.1. Electrical Measurements

As expected, the results showed a gradual decrease of |Z| (Table 1) and phase (φ) (Table 2) with increasing storage times. During post-mortem, a decrease in impedance has been found in the muscles of squid, fish, or various terrestrial animals [15,33,34,35,36]. This is due to a progressive deterioration of the cell membranes and increased conductivity from releasing metabolic products originating from the enzymatic degradation of the muscles. The obtained results concur with our previous findings [15] where low (<5 kHz) and high (>10 MHz) frequencies were not considered because the electrode polarization of those frequencies showed parasitic impedances.

|Z| showed clear visual differences among the groups at all the frequencies below 1 MHz (Figure 2). Nine groups (i.e., days of storage) were clustered into a maximum of six subsets per frequency (Table 1). The impedance magnitude |Z| seems to change more evidently during the first day at T = 4.5 °C. From day 8 until day 11, |Z| was constant, as evaluated at frequencies from 0.05 to 0.10 MHz.

ANOVA and post-hoc Tukey tests indicated that phase (φ) (Table 2) initially had nine groups that were grouped into a maximum of six subsets per frequency. Significant results were obtained for the measurements at 0.5, 1, and 5 MHz. Phase angle (ϕ) measured at 5 MHz was significantly negatively correlated with duration of storage where impedance dropped linearly with the duration, while the samples were grouped into six subsets (Table 2).

For the first day of storage, no linearity between the phase and days of storage was found. The phase was highest for the second day of storage, which was not expected. Again, during the last days of storage, the phase stopped decreasing, but the changes in latter days of storage had more influence on phase than on impedance. A number of studies have been published with similar indications, where impedance is a potential tool for evaluating muscular aging and spoilage in a fast and non-expensive manner for both meat and fish [15,23,37]. However, the potential instrument would need to record the readings at one or a few (preferably) lower frequencies. According to the literature, measurements were taken at a number of frequencies and complex statistical tools were used for interpretation of the results [15,22,23,28,35,36].

**Figure 3:**
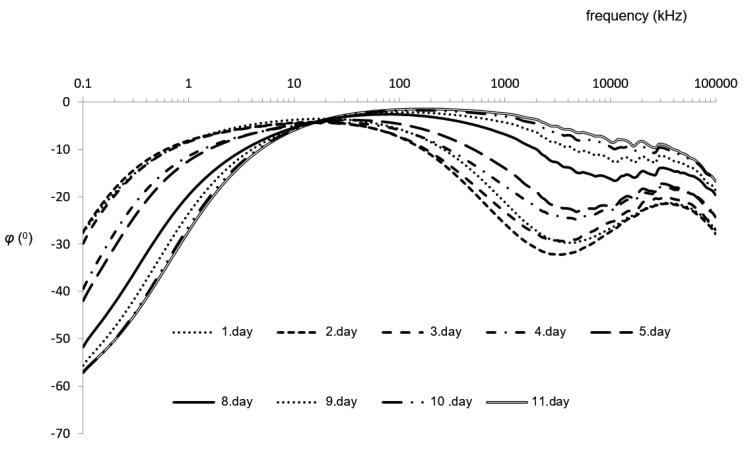
Phase (φ) of European squid samples during chilled storage (4.5 °C, RH 55%) in the entire frequency range.

Interpretation of recordings should be carefully considered, as different factors modify the electrical properties of foods, including moisture, salinity, temperature, structure, electrode configuration, and the interface between electrode and sample [38]. Here fluctuations in moisture, salinity, and temperature are much more reflected in |Z| or resistance than in phase (φ) or the reactance [15,28]. Muscle is electrically anisotropic, meaning that applied high-frequency electrical current flows along, rather than across, muscular fibers. Hence the challenges associated with the orientation of the electrode, where anisotropy can be reduced by the optimal orientation of the electrode, and measuring at higher frequencies that are above the beta dispersion [15]. Anisotropy has also less influence on phase (φ) than on the |Z| [35]. Even though phases recorded at higher frequencies (around 1 MHz) discriminated well between fishes with various freezing histories [33,34,35,36], the properties/histories of the foods should be known when complex impedance is used as a quality assessment during storage. Since impedance is more affected by temperature and position of electrodes than the phase (φ) angle, further discussion will focus on the angle as a potential indicator for the length of storage (Figure 3).

It is common to expect fluctuations in temperature along the seafood chain, so control methods that are independent of temperature are preferable. In a previous study [15] the measurements on chilled squids started on the fourth day post-mortem with phase (φ) measured at 1 MHz, when all the samples were grouped into three groups. The measurements in this study were taken from the first day of capture with the assumption that the samples will be grouped to more than four categories. Therefore, the assessment of storage time after catch should be easier. It was confirmed that changes during chilled storage are reflected in phase (φ) with a broader frequency range of 0.5–10 MHz. Since the evaluation begun on the first day post-mortem, squids were grouped into five groups (days 1–3, days 3–4, days 5–8, days 9–11). However, this interpretation may be challenging, as during the first three days of storage, the phase changed significantly with a lack of the expected linearity. Larger squids (average *m* = 230 g) from our previous study expectedly had longer shelf lives (15 days), accompanied by higher absolute values for impedance |Z| and phase (φ). Therefore, a comparison of the absolute values of the electrical measurements between these two studies is difficult.

Literature implies that impedance spectroscopy successfully predicts the freshness of sea bream (*Sparus aurata*). For filleted samples (*n* = 6 batches), researchers have associated storage time (15 days) with measurements of |Z| and (φ) below 1 MHz. They used chemometric tools such as linear discriminant analysis (LDA) and partial least squares (PLS) to differentiate samples into four groups and according to the days of storage. The experiment started on the first day after fishing when the fish was already in rigor-mortis [23], which might be a potential source of bias. The squid muscle is much more homogenous than the fish counterparts, and post-mortem changes are more uniformed for cephalopods (e.g., rapid changes in proteins); hence, they might be better suited for electrical measurements [15].

### 3.2. Measurements of Color Parameters

The alterations of superficial appearance and coloration in squids were previously identified as a quality indicator [39]. The color of the squids’ skin gradually changes with storage time (Table 3), generating greater redness and lower yellowness. This is consistent with previous studies, where L—value decreased during storage from 43.19 ± 4.44 (day 1) to 27.80 ± 3.76 (day 11), and the samples were grouped into three clusters [15,40]. Furthermore, a*—values increased from 1.53 ± 0.97 (day 1) to 5.84 ± 0.25 (day 11) as shown in Table 3. The most significant changes were obvious for a*—values that grouped samples into six clusters. In our previous study, the most important alterations were also obtained for a*, but here it grouped samples into three clusters. Significant changes were found for b*, which grouped samples into four distinct clusters (days 1−3, days 4−5, days 5−9, and days 10−11). It was previously noticed [15] that the most important changes in color for the parameter b* starts after rigor mortis (e.g., after the fourth day of storage). This observation was confirmed by this study as well. Moreover, PCA analysis with CIELab has already demonstrated that colorimetric evaluation is able to successfully differentiate between fresh (*D. gigas*) and spoiled squids stored at +4 °C for 0, 3, 5, 7, 10, and 12 days [13].

### 3.3. Determination of Total Volatile Nitrogen Base, pH, and Water Holding Capacity

The results of statistical analyses for TVB-N, pH, and WHC are presented in Table 4. The main changes during storage were observed on TVB-N concentrations. The initial TVB-N value was 11.91 ± 0.25 mgN/100 g, presenting slightly lower values than those reported for *Illex argentinus* [41] and *Loligo formosana* [39]. Vaz-Pires et al. [4] detected a TVB-N value of 9.9 mgN/100 g for the 1st day of storage in fresh squid *Illex coindetii*, a similar value as observed on day 2 in the present study. TVB-N levels observed in this study were lower than for other cephalopods, which indicated good quality and freshness [4,39,40,42]. As expected, TVB-N levels significantly increased (*p* ≤ 0.05) by the end of the storage period (Table 4). The values (207.3 ± 0.09 mgN/100g) were highest at day 11. This indicated the existence of nitrogenous constituents in the mantle generated by degradation of (non)proteins by proteolytic bacteria, which instigated a disagreeable odor.

Statistical analysis showed that the samples could be clustered into four distinctive groups during the 11 days of controlled chilled storage (Table 4). Fast increase of TVB-N levels from day 6 probably generated an unpleasant odor that was reported by the sensory panelist. Unpleasant odor was reported on day 11 together with sensory unacceptance by the panelists, which indicated that bacterial activity was increasingly noticeable. The overall results show that TVB-N levels are a good indicator of deterioration, but daily checks are necessary for accurate assessments of spoilage.

TVB-N assessments are relatively simple, but they tend to identify advanced stages of spoilage only. Hence, they are widely considered as unreliable for the first ten days of the chilling of cod and other species [43]. As previously reported, TVB-N levels tend to increase with storage time in squid samples [39,40,42]. Huss (1999) [44] stated that TVB-N content is dependent, for example, on the concentration of trimethylamine N-oxide (TMAO) in muscles, which further depends on the genetic factors, size, feeding, fishing zone, season, and microbiological load. Since TMA is a function of bacterial activity, it, therefore, contributes to the TVB-N value [42].

The WHC is yet another quality parameter that decreases during storage, as observed by other decreases of the chemical and structural properties of muscular tissues as well as post mortem transformations. As expected, WHC was highest at the beginning of storage and the lowest on day 11. It was previously reported that WHC decreases with an increase in temperature and denaturation of proteins [45]. Initial WHC was found to be 37.07 ± 4.46 and the value significantly dropped to 7.42 ± 0.01 (*p* ≤ 0.05) after 11 days of storage (Table 4). Typically, protein status and degradation of myofibrillar proteins are directly related to the WHC of fish and cephalopods; hence, it is meaningful to explain the increase in TVB-N levels and pH values with the decrease in WHC. Statistical results on WHC confirmed that the samples could be clustered into three distinctive groups during the 11 days of storage (Table 4).

pH values increase from 6.66 ± 0.08 on the second day of storage to 7.42 ± 0.01 on day 11. During the first day, accumulated lactic acid from glycolysis initiates a reduction in pH, which normally promotes the rigor mortis, as previously reported for fresh squid [14,46]. The increase in storage temperature is a further reason for the observed increase in the pH of the mantles. Considering pH values, the samples can be clustered into three groups (Table 4).

### 3.4. Correlations Between Measured Parameters

Correlations from Table 5 show coefficients for different variables that were selected based on Pearson’s coefficients and the results obtained in this study. The relevant rapid technique for inexpensive evaluation of freshness can be evaluated by CIELab parameters [13,39]. In a previous study, we have reported that the phase angle (ϕ) measured at 1 MHz was somewhat correlated with the length of storage in comparison to a*-values [15]. This was confirmed in the current study, although the duration of chilled storage had the strongest correlation with phase angle (ϕ), measured at 5 MHz for smaller samples. Current and previous reports related to squids were similar to those reported for fish [35]. It is obvious that both the length of storage and the EU scheme have very strong positive correlations with phase (ϕ) at 5 MHz. Phase ϕ at 5 MHz is highly positively related with a*, b*, TVB-N, and highly negatively correlated with the WHC. Similarly, the length of chilled storage negatively associates with the WHC, but all other quality parameters increase with time (Table 5).

In particular, it should be emphasized that the phase (ϕ) depends on the inner characteristics of the samples, therefore presented results are applicable to the squids *L. vulgaris* of similar dimensions and chemical composition. Squids do not have significant annual differences in chemical composition, but differences due to length could be significant. However, as pointed out before, the phase (ϕ) is still promising parameter as an indicator of quality in biological tissues, as it is less affected by external factors such as temperature and position of electrodes. On the other hand, impedance |Z| is biased by external factors (e.g., ambience and sample temperature); thus impedance measurements were not included in Table 5.

### 3.5. Sensory Evaluation of the Raw Squid

With a decline of freshness, fish appearance, taste, flavor, and texture are prone to changes [47]. This study employed the official method for the assessment of the quality of fishery products in Europe [32]. The EC scheme clustered the samples into four groups (E, A, B, and unacceptable). Post-mortem changes during storage showed that maximum freshness was obtained between the first and third days of storage (E), while medium freshness was observed between the fourth and fifth days (A).

A significant increase of TVB-N levels on day 6 generated unpleasant odors that was noticed by the sensory panel (Table 6). The samples became unacceptable on day 10. Sensory evaluation of raw L. *vulgaris* showed freshness was lowest between days 8−10 of storage (B). It was suggested that the samples (average *m* = 130 ± 20 g) from the first till the eighth day after capture were fit for consumption or processing. Association of a phase angle (ϕ) with the length of storage increased in strength with passage of time, hence caused better groupings of samples, i.e., after the rigor mortis, there was a clearer linear drop in the phase angle (ϕ) with longer storage. So even though samples were compliant with the EC scheme in the first five days of storage, they were better differentiated by the electrical measurements in the latter days.

## 4. Conclusions

The study shows that impedance spectroscopy is able to give information about the storage time (4.5 °C, 55% relative humidity) after a catch of European squid (*L. vulgaris*). This fast and non−destructive method, coupled with affordable instrument design, has the potential as a control system for the rapid storage-time assessments of cephalopods by measuring phase at frequencies around 5 MHz. This work contributes to the advance of rapid and cheap methods based on bioelectrical impedance to gauge cephalopod quality with respect to colorimetric parameters, sensory properties, total volatile nitrogen, pH, and water holding capacity. However, further studies will be necessary to address the influence of squid size on the proposed electrical measurement.

Currently, no single instrumental method that can determine the freshness of all fish or cephalopods exists. It is important to develop sensory methods for *Loligo vulgaris*, to have a foundation for comparison of the results with the phase angle results. In that way, data will be more useful for the industry, since phase angle measurements are independent of the ambient temperature and samples’ temperature. It is evident that the sample size has a significant influence on the results of phase angle and impedance, and a shorter storage time at lower air temperature is possible.

## Figures and Tables

**Figure 2:**
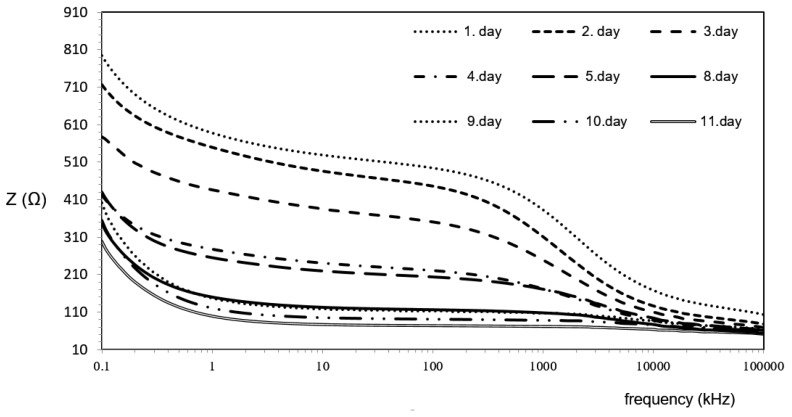
Impedance |Z| measurements of European squid samples during chilled storage (4.5 °C, RH 55%) in the entire frequency range.

**Table 1:** Impedance magnitude (|Z|) of European squid (*Loligo vulgaris*) measured at selected frequencies during storage at 4.5 °C and 55% of relative humidity.

Day of Storage	Z 5 kHz	Z 10 kHz	Z 100 kHz	Z 1 MHz
1	541.16 ± 69.823 a	528.83 ± 67.92 a	494.33 ± 62.16 a	378.29 ± 45.79 a
2	500.75 ± 67.60 a	486.13 ± 65.10 a	446.53 ± 57.8 b	306.77 ± 32.11 b
3	398.01 ± 81.68 b	384.72 ± 77.30 b	350.95 ± 69.04 c	246.15 ± 43.45 c
4	248.96 ± 60.93 c	240.41 ± 59.67 c	220.72 ± 54.83 d	170.05 ± 39.46 d
5	227.05 ± 28.27 c	219.74 ± 27.15 c	203.99 ± 24.68 d	170.18 ± 20.35 d
8	127.41 ± 17.50 d	123.05 ± 17.16 d	116.74 ± 16.47 e	108.12 ± 15.15 e
9	122.21 ± 14.55 d	118.19 ± 13.74 d	112.64 ± 12.63 e	106.99 ± 11.58 e
10	98.22 ± 23.21 d	95.04 ± 22.71 d	90.98 ± 21.81 e	87.84 ± 21.39 ef
11	80.33 ± 20.07 d	77.67 ± 19.51 d	74.63 ± 18.73 e	72.51 ± 18.39 f

Different letters in the same column indicate a significant difference among different days of storage (*p* ≤ 0.05).

**Table 2:** Phase (φ) of European squid (*Loligo vulgaris*) measured at selected frequencies during storage at 4.5 °C and 55% of relative humidity.

Day of Storage	φ 5 kHz	φ 100 kHz	φ 500 kHz	φ 1 MHz	φ 5 MHz	φ 10 MHz
1	−4.34 ± 0.31 ^a^	−5.65 ± 0.77 ^b^	−15.1 ± 1.93 ^b^	−21.63 ± 2.04 ^b^	−29.5 ± 1.42 ^ab^	−26.63 ± 1.13 ^a^
2	−4.99 ± 0.29 ^ab^	−7.41 ± 0.71 ^a^	−19.39 ± 1.68 ^a^	−26.09 ± 1.95 ^a^	−31.23 ± 1.42 ^a^	−27.22 ± 1.136 ^a^
3	−5.10 ± 0.76 ^ab^	−7.3 ± 0.91 ^a^	−16.82 ± 1.71 ^a^	−22.54 ± 1.96 ^a^	−28.7 ± 2.03 ^b^	−25.30 ± 1.73 ^a^
4	−6.07 ± 1.45 ^b^	−5.8 ± 0.75 ^b^	−13.41 ± 1.84 ^c^	−18.0 ± 2.39 ^c^	−24.75 ± 2.33 ^c^	−22.65 ± 1.71 ^b^
5	−6.02 ± 0.83 ^b^	−4.53 ± 0.48 ^c^	−10.13 ± 1.04 ^d^	−14.21 ± 1.29 ^d^	−22.89 ± 1.66 ^c^	−21.92 ± 1.52 ^b^
8	−7.88 ± 1.31 ^c^	−2.6 ± 0.44 ^d^	−4.87 ± 1.01 ^e^	−6.97 ± 1.41 ^e^	−14.99 ± 2.08 ^d^	−16.28 ± 1.82 ^c^
9	−8.47 ± 0.80 ^cd^	−2.08 ± 0.3 ^de^	−3.16 ± 0.79 ^f^	−4.32 ± 1.18 ^f^	−10.43 ± 1.92 ^e^	−12.18 ± 1.80 ^d^
10	−9.19 ± 1.63 ^d^	−1.76 ± 0.36 ^e^	−2.29 ± 0.79 ^f^	−3.07 ± 1.08 ^f^	−8.08 ± 1.50 ^f^	−9.55 ± 1.67 ^e^
11	−9.55 ± 1.58 ^d^	−1.57 ± 0.36 ^e^	−1.88 ± 1.09 ^f^	−2.50 ± 1.74 ^f^	−6.53 ± 3.29 ^f^	−8.54 ± 3.21 ^e^

Different letters in the same column indicate a significant difference among different days of storage (*p* ≤ 0.05).

**Table 3:** Color parameters of European squid (*Loligo vulgaris*) after different days of storage at 4.5 °C and 55% of relative humidity.

Day(s) of Storage	L*	a*	b*
1	43.19 ± 4.44 ^a^	1.53 ± 0.97 ^a^	−1.18 ± 0.98 ^a^
2	40.07 ± 5.07 ^a^	1.78 ± 1.12 ^ab^	−1.31 ± 1.02 ^a^
3	35.73 ± 3.58 ^b^	2.22 ± 0.79 ^abc^	−1.63 ± 0.46 ^a^
4	32.37 ± 3.07 ^bc^	2.95 ± 1.15 ^bcd^	1.47 ± 1.24 ^b^
5	31.61 ± 2.73 ^bc^	3.45 ± 1.13 ^cde^	2.35 ± 0.71 ^bc^
8	30.41 ± 3.06 ^c^	3.86 ± 0.95 ^de^	2.87 ± 1.19 ^cd^
9	29.39 ± 4.05 ^c^	4.62 ± 1.12 ^ef^	2.92 ± 0.49 ^cd^
10	28.97 ± 3.50 ^c^	5.35 ± 1.70 ^f^	3.63 ± 1.16 ^d^
11	27.80 ± 3.76 ^c^	5.84 ± 1.25 ^f^	3.95 ± 1.11 ^d^

Different letters in the same column indicate a significant difference for different days of refrigerated storage (*p* ≤ 0.05). L*—lightness; a*—redness; b*—yellowness.

**Table 4:** Total volatile base nitrogen (TVB-N), water holding capacity (WHC), and pH values of European squid (*Loligo vulgaris*) during storage at 4.5 °C and 55% of relative humidity.

Day of Storage	TVB−N (mg N/100 g)	WHC	pH
2	11.91 ± 0.25 ^a^	37.07 ± 4.46 a	6.66 ± 0.08 ^a^
5	16.12 ± 0.12 ^b^	15.94 ± 0.53 b	6.96 ± 0.21 ^a^
9	63.03 ± 0.18 ^c^	13.76 ± 0.00 c	7.35 ± 0.15 ^b^
11	207.3 ± 0.09 ^d^	12.34 ± 0.00 c	7.42 ± 0.01 ^c^

Different letters in the same column indicate a significant difference among different days of storage (*p* ≤ 0.05).

**Table 5:** Pearson coefficients calculated between the days of storage at 4.5 °C and 55% of relative humidity, EC, ϕ at 5 MHz, a*, b*, WHC, and TVB-N.

	Days	EC	ϕ ατ 5 ΜHζ	a*	b*	WHC	TVB-N
Days	1	0.956 *	0.968 *	0.785 *	0.845 *	−0.824 *	0.848 *
EC		1	0.935	0.779 *	0.875 *	−0.831 *	0.894 *
ϕ ατ 5 ΜHζ			1	0.754 *	0.810 *	−862 *	0.768 *
a*				1	0.651 *	−0.584 *	0.860 *
b*					1	−0.925 *	0.609
WHC						1	−0.546 *
TVB-N							1

* Correlation is significant at *p* ≤ 0.05; EC—EC scheme; at 5 MHz—phase angle on 5 MHz; a*—redness; b*—yellowness; WHC—water holding capacity; TVB-N—Total Volatile Base Nitrogen.

**Table 6:** Results of sensory evaluation for row squids (*Loligo vulgaris*).

Measurements	Storage Days	Scores According to Kumar et al. [48]	Category According to EC Scheme	Groups
1	0.5	10	E	1
2	2	10	E	1
3	3	10	E	1
4	4	9	E	1
5	5	8	A	2
6	8	7	A	2
7	9	6	B	3
8	10	5	B	3
9	11	4	B	3
10	12	1	C	4
